# Blurred Palmprint Recognition Based on Stable-Feature Extraction Using a Vese–Osher Decomposition Model

**DOI:** 10.1371/journal.pone.0101866

**Published:** 2014-07-03

**Authors:** Danfeng Hong, Jian Su, Qinggen Hong, Zhenkuan Pan, Guodong Wang

**Affiliations:** 1 College of Information Engineering, Qingdao University, Qingdao, China; 2 School of Communications and Information Engineering, University of Electronic Science and Technology of China, Chengdu, China; 3 Research Institute of higher education, Department of Training, Naval Aeronautical and Astronautical University, Yantai, China; Institute of Automation, Chinese Academy of Sciences, China

## Abstract

As palmprints are captured using non-contact devices, image blur is inevitably generated because of the defocused status. This degrades the recognition performance of the system. To solve this problem, we propose a stable-feature extraction method based on a Vese–Osher (VO) decomposition model to recognize blurred palmprints effectively. A Gaussian defocus degradation model is first established to simulate image blur. With different degrees of blurring, stable features are found to exist in the image which can be investigated by analyzing the blur theoretically. Then, a VO decomposition model is used to obtain structure and texture layers of the blurred palmprint images. The structure layer is stable for different degrees of blurring (this is a theoretical conclusion that needs to be further proved via experiment). Next, an algorithm based on weighted robustness histogram of oriented gradients (WRHOG) is designed to extract the stable features from the structure layer of the blurred palmprint image. Finally, a normalized correlation coefficient is introduced to measure the similarity in the palmprint features. We also designed and performed a series of experiments to show the benefits of the proposed method. The experimental results are used to demonstrate the theoretical conclusion that the structure layer is stable for different blurring scales. The WRHOG method also proves to be an advanced and robust method of distinguishing blurred palmprints. The recognition results obtained using the proposed method and data from two palmprint databases (PolyU and Blurred–PolyU) are stable and superior in comparison to previous high-performance methods (the equal error rate is only 0.132%). In addition, the authentication time is less than 1.3 s, which is fast enough to meet real-time demands. Therefore, the proposed method is a feasible way of implementing blurred palmprint recognition.

## Introduction

Biometrics attempts to effectively verify the identity of a living person using physiological or behavioral characteristics. Techniques include face [1]–[3], fingerprint [4]–[8], and palmprint recognition [9]–[18], etc. As an important biological characteristic, palmprint-based recognition has attracted much attention due to its various merits, e.g. high accuracy, low-cost, and easy availability. In the past few decades, palmprint recognition has become an emerging technology in the field of biometric identification and some very fruitful research achievements have been obtained [9]. According to the representation method of palmprint features, they can be classified into three main categories: principal line extraction [10]–[12], subspace learning [13]–[14], and texture coding [15]–[17]. Although these methods are capable of high recognition accuracy, they are usually based on contact devices being used to capture the palmprint image, e.g. scanners, CCD devices, etc. [15]. These collection methods may cause some trouble for the user, for example, sanitary and psychological problems. However, the most crucial problem is that the palmprint template is liable to become lost, which can make the recognition system permanently invalid.

An effective solution to these problems is to use non-contact devices [19]–[21] in place of the contact ones. However, unlike contact methods, non-contact devices will inevitably suffer from blurred images. Blurring degrades the image quality, making it difficult to extract distinctive features from the image. Thus, blurred-image recognition is a novel problem in biometrics. It is associated with some tough problems, e.g. difficult feature extraction, loss of texture information, etc. As a result, many of the previous recognition methods do not work.

Some solutions to address these issues have been proposed. Yuan et al. [21] designed a simulation system for non-contact online palmprint recognition incorporating the constraints of the collection device. An evaluation standard was used to measure image definition to select a satisfactory palmprint image. Nevertheless, the low image acquisition speed and lack of robustness make the method unsuitable for large palmprint databases and real-time applications. Image blur can also be solved from the perspective of image processing, e.g. restoration or enhancement. Kang et al. [22] utilized template convolution to calculate the focus value of an image. A least-square method was used for image restoration by setting a threshold value for the focus value. This method has been successfully applied in iris recognition. Cheng et al. [23] developed an image enhancement algorithm based on orientation fields to effectively improve the image quality.

Although image restoration and enhancement technologies can improve image quality, these methods are, unfortunately, complex and so they are a burden to use in the field. Besides this, it is difficult to find a universal standard to measure the quality of different images. This is because the reason for and the scale of the blurring differ from image to image. Therefore, blurred palmprints are undesirable for use in recognition using image processing technology. In view of the above problems, some scholars have suggested that there are stable features in palmprint images suffering from different levels of blurriness. If so, directly using these stable features in blurred palmprint images in the recognition process could become an effective solution for blurred palmprint recognition. Sang et al. [13] achieved blurred palmprint recognition using two-dimensional principal component analysis (2DPCA). The data obtained using 2DPCA can be viewed as the stable features required. According to analysis of the experiment results, the idea proved to be effective. However, recognition accuracy is not particularly high due to a lack of distinctive features in the 2DPCA procedure. On the basis of Ref. [13], Lin et al. [14] used a Laplacian smoothing transformation (LST) in place of 2DPCA to improve recognition accuracy. The results obtained were indeed better. In Ref. [11], Lin et al. extended their work and proposed a new blurred-palmprint recognition method DCT–BEPL based on discrete cosine transformation (DCT) and block energy of principal lines (BEPL). Their work proved that orientation features also form stable information in blurred palmprint images. As a result, the recognition results were improved once again compared with LST. However, there is much scope for further improvement in the aspect of recognition accuracy because their methods did not have a specific fusion rule in them.

The research presented here, based on a theoretical analysis of the above existing methods, concludes that an effective method for blurred palmprint recognition is to extract stable features from the blurred image (containing principal components and orientation information). We further propose a new method which takes full advantage of the importance of the stable features in blurred palmprint recognition. We first extract the structure layer of the blurred palmprint image using a Vese–Osher (VO) decomposition model. The structure layer is stable with respect to different degrees of blurring. Secondly, to extract the distinctive orientation features from the structure layer, we improve the histogram of oriented gradients (HOG) method (a desirable descriptor of the direction characteristics). In order to further improve recognition accuracy, a fractal weight is added to the improved HOG method. The proposed method is referred to as the ‘VO–WRHOG’ method. Finally, we used a normalized correlation coefficient (NCC) to measure the similarity between the feature vectors. Compared with previous high-performance palmprint recognition methods, the recognition results obtained using the VO–WRHOG method are more stable and effective (as applied to two databases: one containing clear palmprints and the other blurred palmprints). Furthermore, the recognition accuracy is higher than that using the previous methods for the blurred palmprint database. In addition, the proposed method is fast enough to meet the demands required for real-time application.

The remainder of this paper is organized as follows: Section 2 introduces the background knowledge and methods. Section 3 introduces the VO–WRHOG method to extract the stable features from blurred palmprint images and briefly describes the feature-matching method. Section 4 reports the experimental results and analysis. Section 5 contains a discussion and our conclusions, as well as the future prospects of the method.

### Ethics statement

The PolyU palmprint database used in this paper is publicly available and can be downloaded from the website of the Biometric Research Centre (UGC/CRC) at the Hong Kong Polytechnic University. It is totally free for academic and noncommercial use. We have been allowed to download the PolyU palmprint database by filling in the agreement forms at the website of the UGC. Finally, the palmprint images and experimental results are reported only for academic research without any commercial use.

## Related Knowledge and Methods

### 2.1 Preprocessing palmprint images

**Figure 1:**
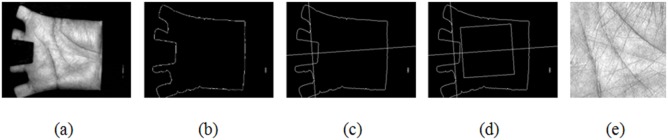
Illustration of palmprint image preprocessing.

Image preprocessing is a prerequisite for image retrieval and recognition, especially for palmprints [15] and fingerprints [6]. Recognition results can be substantially improved via the preprocessing procedure which can deal with many troubling problems, e.g. rotation and translation alignment, region of interest (ROI), and so forth. In this paper, the PolyU palmprint database is used as test material for our method. The palmprint images are captured by a CCD device [17], which can remove rotation and translation phenomena to some extent. In addition, in Ref. [15], a segmentation method was proposed for palmprint images captured using CCD devices to obtain the regions of interest. Thus, these methods are directly used in our paper. Fig. 1 shows an illustration of palmprint image preprocessing. The obtained ROI contains 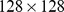 pixels with 256 gray levels per pixel.

### 2.2 Image blur theory

**Figure 2:**
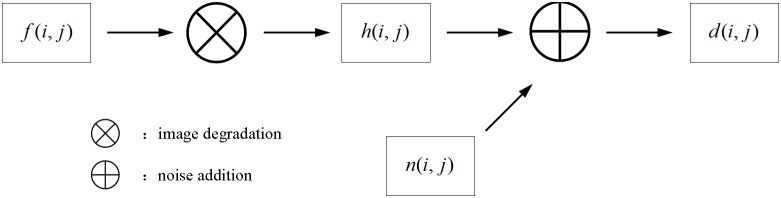
The image degradation model.

Image blur can be considered to be equivalent to having a clear, non-distorted image convoluted with a degradation function in the spatial domain. The general concept is illustrated in Fig. 2. If the image remains space-invariant in the process of degradation, we can write
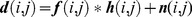
(1)where 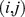 denotes the position in the image, 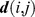 the blurred image, 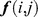 the non-blurred image, 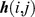 the degradation function, and 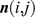 is additive noise. The operator ‘
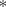
’ denotes convolution.

**Figure 3:**
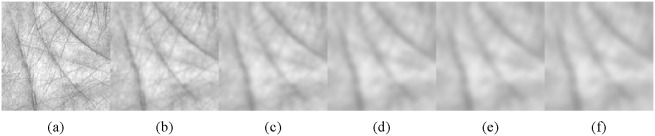
The ROI with different degrees of blurring (*σ*). (a)–(f) correspond to the ROI with blurring set to 1, 2, 3, 4, 5, and 6, respectively.

**Figure 4:**
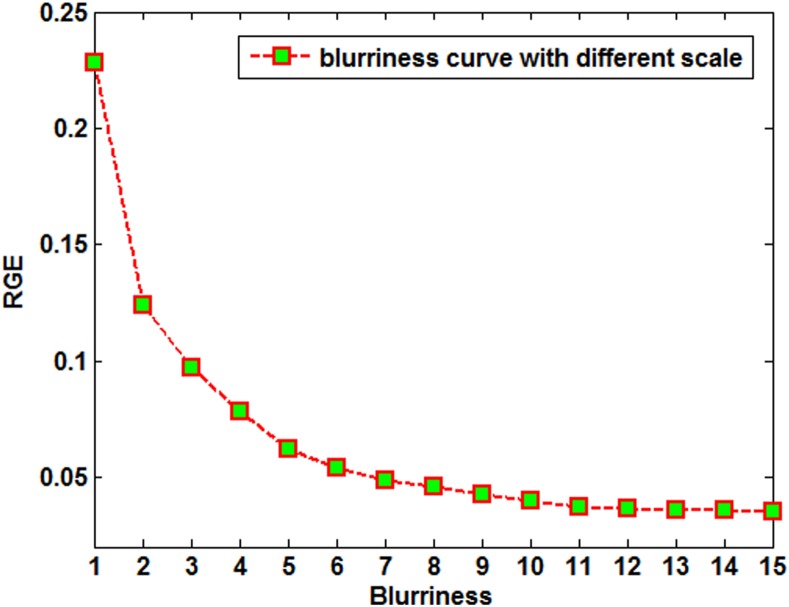
Blurriness curves corresponding to Fig. 3.

We can see from Fig. 2 and Eq. (1) that 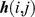 plays an important role in the process of degradation. There are many kinds of degradation function and in Ref. [24] Wang et al. list several of the more common ones. The Gaussian defocus degradation model (GDDM) is one of the most effective models for simulating image blur, as proved by a large number of researchers. GDDM is expressed using the following equation:
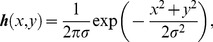
(2)where 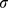 is the sampling width of the filter which controls the degree of image degradation. As the value of 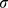 increases, image degradation becomes more obvious and the image becomes more blurred. In Ref. [25], several methods of evaluating blur are defined which have proved to be effective and correct. Here, in order to clearly show the relationship between blurriness and 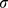, we select one of those effective methods to use as our evaluation standard, the Robert gradient energy (RGE). The definition of this measure is described as follows
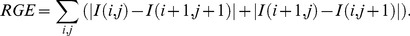
(3)Here, 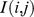 stands for the gray value of the image at position 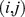. The RGE value reflects the definition of the image. As the RGE value increases, the image becomes sharper. Conversely, if the RGE value wanes, the image becomes more blurred. If we use the GDDM with different 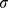 values to simulate image blur, the corresponding RGE values can be obtained to reflect the degree of blurriness. Fig. 3 shows a ROI with different degrees of blurring (i.e. different 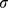 values) and Fig. 4 shows the blurriness curve corresponding to Fig. 3.

In Fig. 4, as the 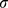 value varies from 1 to 15, the RGE value becomes smaller. However, when the 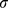 value is greater than 10, the RGE value seems to be constant. Therefore, we can use the 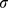 value (e.g. in the range from 1 to 10) to represent the blurriness of an image. We considered this to be an approximate standard by which to measure the level of blur in a palmprint in this paper.

### 2.3 VO decomposition model

Meyer pointed out that an image can be divided into a structure layer and a texture layer using image decomposition. This can be expressed as
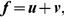
(4)where ***f*** is the original image, and ***u*** and ***v*** are the structure and texture layers of the image, respectively. On this basis, Meyer [26] introduced the concept of a *G* space which is substituted for the *L* ^2^-norm used in the total variation (TV) model to describe the oscillating component of the image. This established the total variation based on *G* space (TV–G) model for image decomposition applications. The TV–G model is defined as follows



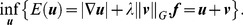
(5)In Eq. (5), the oscillating component of the image (which contains texture and noise) is described using the *G* space.

Meyer did not propose a method for solving the corresponding TV–G model. Therefore, based on Meyer’s thoughts, many numerical methods were proposed to solve the TV–G model. The results proved that *G* space is effective for describing the oscillating component of images. Among these methods, Vese and Osher [27] established a decomposition model for approximating the solutions to Meyer’s theory. The VO model is defined as
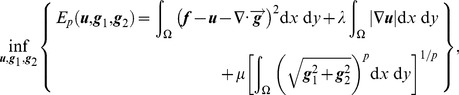
(6)where 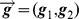 and 
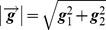
. The texture layer ***v*** is expressed via 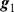 and 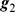 using 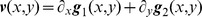. The quantities 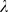 and 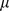 are positive penalty parameters which are used to adjust the proportions of the different types of information in the model. Based on a large number of experimental results, Vese and Osher reached the conclusion that the performance of the VO model is the optimal when the parameter *p* is set equal to 1. In this case, Eq. (6) can be rewritten as



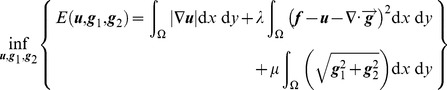
(7)Eq. (7) is a multivariate minimization problem and is usually solved using a variational method [28]. Thus, we put forward the Euler–Lagrange equations
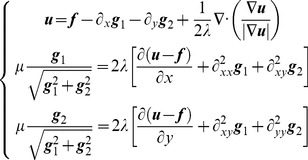
(8)


In addition, the boundary constraints corresponding to Eq. (7) are
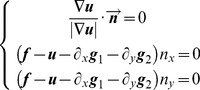
(9)where 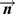 is normal vector of the boundary and 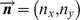.

We consider the gray value of each pixel as the sampling value of ***u*** and use the finite difference method to solve Eqs. (8) and (9) via an alternating optimization procedure [28]. First, we fix ***g*** to optimize ***u***. Then, we fix ***u*** while optimizing ***g***. Finally, we obtain the structure layer ***u*** and texture layer ***v***.

In Meyer’s image decomposition theory [25], the properties of the structure layer obtained using the VO decomposition model were summarized as follows. (i) The structure layer contains the primary component of the image. This layer has properties similar to the low frequency information obtained via frequency domain analysis. (ii) The structure layer is barely affected by noise or blurred textures. (iii) The structure layer can maintain the spatial organization of the original image (perhaps, the most important property).

**Figure 5:**
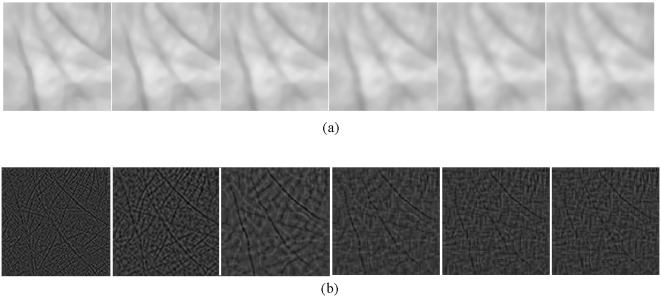
Structure and texture layers with different degrees of blurring corresponding to Fig. 3 obtained using the VO decomposition model. (a) is the structure layer with different degrees of blurring, and (b) the corresponding texture layer.

**Figure 6:**
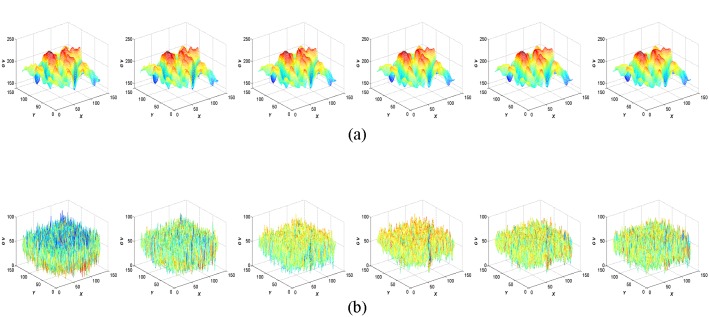
Surface plots of the structure and texture layers corresponding to Fig. 4 for: (a) the structure layer, and (b) the texture layer.

Fig. 5 shows the structure and texture layers of Fig. 3 obtained using VO decomposition. We can see from the figure that the structure layer basically remains unchanged regardless of the degree of blurring. Also, the texture layer, which becomes unstable, is damaged because of image blur. Fig. 6 shows surface plots of the structure and texture layers corresponding to Fig. 5 (*GV* stands for gray value). We can see more clearly from Fig. 6 that the structure layer is more stable than the texture layer with respect to the different blurring scales. Therefore, according to Meyer’s theory, and supported by a VO decomposition example, we conclude that theoretically, even if an image suffers from different amounts of blurring, the structure layer of the image remains stable. To make our theoretical conclusion more convincing, we performed related experiments to further illustrate the correctness of the theory (*vide infra*).

## The WRHOG Method

In this section, a weighted robustness histogram of oriented gradients (WRHOG) method is proposed in order to further extract the stable features from the structure layer of the blurred palmprint image. We divide the section into three parts to describe the WRHOG method in some detail.

### 3.1 Histogram of oriented gradients

Using a scale-invariant feature transformation [29], Dalal et al. [30] presented a histogram of oriented gradients (HOG) for detection of humans. HOG is a spatial descriptor and is highly capable of describing orientation features.

First, an orientation map of the palmprint image is obtained via the gradient operator, which is defined using
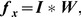
(10)

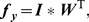
(11)

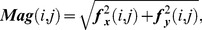
(12)

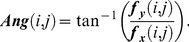
(13)Here, ***I*** stands for the original image (size 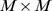) and ***W*** = [–1,0,1] is a convolution mask. The quantities 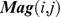 and 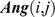 are the gradient magnitude and angle of 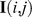, respectively, where 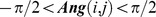. The quantity ***Ang*** is considered to be the orientation map of ***I***. There are many methods available for computing the orientation map [4]–[7]. Jain et al. [4] proposed a method of estimating the orientation map by using gradient. When we computed our orientation maps, we distinguished between two concepts, namely, the gradient vector orientation (GVO) and image orientation (IO) [7]. The scope of the argument in the GVO concept is 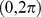; for IO it is 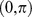. We used GVO as the orientation map in our proposed method because we need to obtain the HOG information. Therefore, we transform 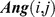 from 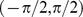 to 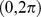. The orientation map is therefore rewritten as follows



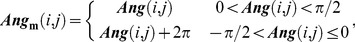
(14)

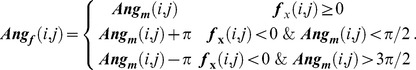
(15)



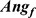
 is the updated orientation map obtained via Eqs. (14) and (15).

Next, the HOG is formed using 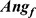, including *N* bins covering the 2π range of orientation. Each pixel added to the histogram is weighted using 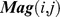. The HOG is obtained as follows
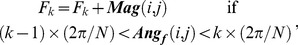
(16)where 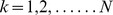. The 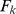 values are usually normalized in order to improve the robustness with respect to illumination and noise, that is, 
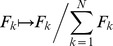
. Therefore, the HOG feature is formulated as
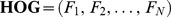
(17)


### 3.2 Robust histogram of oriented gradients

**Figure 7:**
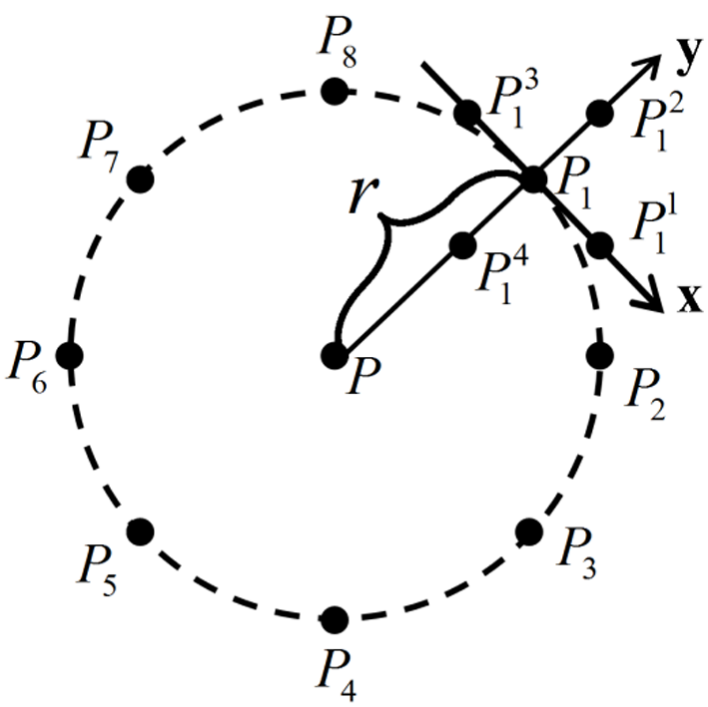
Sketch of the rotation-invariant gradient operator.

When the palmprint image is collected, translation and rotation problems are often encountered. Although these problems can, to some extent, be corrected via preprocessing [15], there still exists a slight offset. HOG information obtained using the traditional gradient operator (Eqs. (10) and (11)) has been well applied in human detection. However, due to the limitations of the traditional gradient operator, poor rotational invariance makes the HOG unstable for palmprint recognition. In Fig. 7, *P* is a pixel point at the center of a circle with radius *r* and 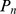 is a sample point on this circle (the number of sample points is 8 and *r* is 2 in this paper). Then, as shown Fig. 6, a local *x–y* coordinate system can be established using *P* and 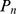 for each sample point. It is clear that we can obtain a rotation-invariant gradient operator (RIGO) in such a coordinate system. The RIGO for point *P* can be computed as follows
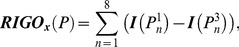
(18)

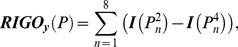
(19)where 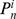 (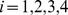) are the surrounding points 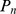 along the *x* and *y* axes, and 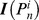 is the gray value at 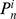.

Therefore, the rotation-invariant histogram of oriented gradients (RIHOG) is obtained by replacing the traditional gradient operator in HOG with the RIGO. Eq. (17) is thus rewritten as
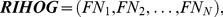
(20)where 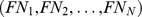 stands for the updated 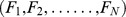 quantities obtained using the RIGO.

The palmprint images are divided into non-overlapping blocks of 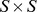 pixels to reduce interference from translation and improve the distinguishability of features – then the RIHOG data is extracted from each block. Finally, a robust histogram of oriented gradients (RHOG) is obtained by combining the RIHOG of each block together. Thereby, the palmprint image is divided into 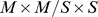 blocks, and the RHOG is expressed as
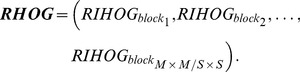
(21)


### 3.3 Fractal dimension

Fractal dimension [31] is usually used to measure the regularity of an object’s surface. If the palmprint image is considered as a surface in three-dimensional space, its fractal dimension can achieve better results in describing the structural features of the image. Hence, we combine fractal dimension with RHOG to obtain a superior descriptor for the palmprint image. There are several methods of calculating fractal dimension. Among these methods, the differential box counting method [32] is one of the most effective methods. A description of the method follows.

An image of size 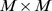 is divided into non-overlapping grids, each of 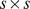 pixels, where *s* is the current scale of the image. Consider the image to be a three-dimensional space in the form 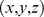, where 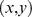 stands for a point in the plane of the coordinate system, and *z* corresponds to the gray value at position 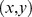. The grid is filled using 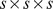 sized boxes. If the minimum and maximum gray value of each grid is located in the *h-*th box and *l-*th box, respectively, the total number of boxes in the grid is
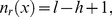
(22)where 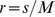. The total number of boxes in the image is
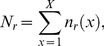
(23)where 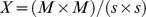 is the quantity of grids. Hence, the fractal dimension *D* is obtained using



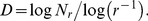
(24)Likewise, palmprint images are divided into non-overlapping blocks of 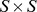 pixels, and the fractal dimension of each block computed to use as weights in the RHOG.

**Figure 8:**
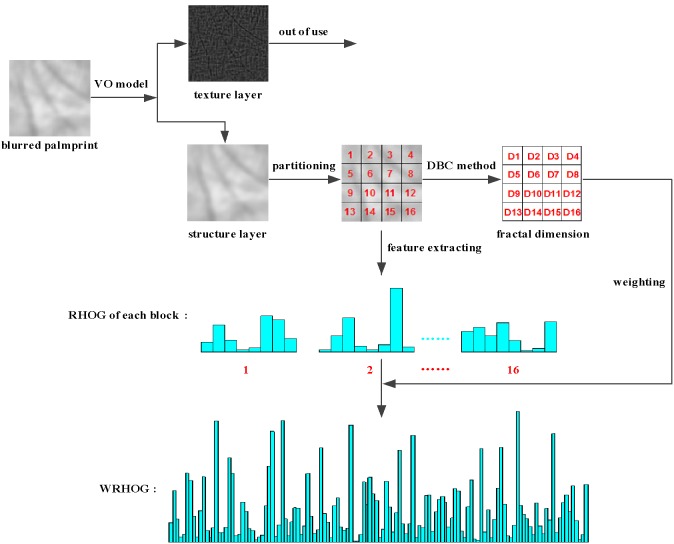
Outline of the proposed VO–WRHOG method.

We obtain the WRHOG for extracting the stable features from the structure layer of a blurred palmprint image via the three steps above. The form of the WRHOG is defined as follows
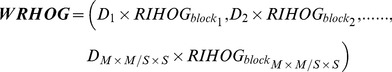
(25)where 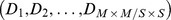 are the fractal dimensions corresponding to each block of the image. The outline of the proposed VO–WRHOG method is as shown in Fig. 8.

### 3.4 Feature matching

A normalized correlation coefficient [18] is used in this paper to scale the comparability between palmprint features. Suppose that 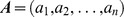 and 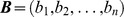 are two vectors of the WRHOG. Then, their NCC is defined as
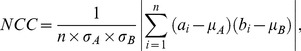
(26)where 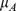 (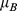) is the mean of ***A*** (***B***), 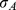 (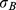) is the standard deviation of ***A*** (***B***), and *n* is the length of ***A*** or ***B***. The value of NCC is between –1 and 1. If NCC is close to 1, it means the palmprint image largely results from the same one; otherwise, it is more likely to be different from one. Hence, the features can be effectively classified by setting a threshold value.

## Experiment and Analysis

In this section, all the experiments performed involve the PolyU palmprint database [15], [33], which includes 7752 palmprint images captured from 386 different palms. Samples from each of the palms were collected in two separate sessions. The average time interval between the two sessions was two months (10 samples were captured in both the first and second sessions). With the intention of verifying the effectiveness of the proposed method, we used two different palmprint databases to test our method. One is the PolyU palmprint database. The other is a blurred PolyU palmprint database which was obtained from the PolyU palmprint database using GDDM with random degrees of blurring (as in Eq. (2)). For the two palmprint databases, the gallery sets were both extracted from the first session. The probe sets were both extracted from the second session. Each image in the probe set is matched with all the images in the gallery set. If the two palmprint images are from the same palm, a genuine match is counted; otherwise, an imposter match is registered. Therefore, the total number of matches is 14,899,600. Among them, there are 38,600 genuine matches and the rest are imposter matches. False rejection and false acceptance rates (FRR and FAR, respectively) are used as evaluation standards and are defined by
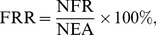
(27)

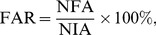
(28)where NEA (number of enrollee attempts) and NIA (number of impostor attempts) represent the true and false matching numbers, respectively. NFR (number of false rejections) and NFA (number of false acceptances) are the numbers of false rejections and false acceptances, respectively.

A receiver operating characteristic (ROC) curve, which is constructed using FAR and FRR data, is usually used to measure the performance of the algorithm. When the value of the FAR is equal to FRR, the FAR value (or FRR) is called the equal error rate (EER). The lower the value of EER, the better the performance of the algorithm. We carried out five experiments to evaluate the performance of our algorithm, and to demonstrate that the proposed method is effective and convincing. These tests concerned selection of the optimal parameters for the VO–WRHOG method, consideration of the stability of the structure layer, the benefits of the proposed method, a performance comparison with traditional algorithms, and a consideration of the algorithm’s relative speed, respectively, as we discuss below.

### 4.1 Optimal parameters for the VO–WRHOG method

**Figure 9:**
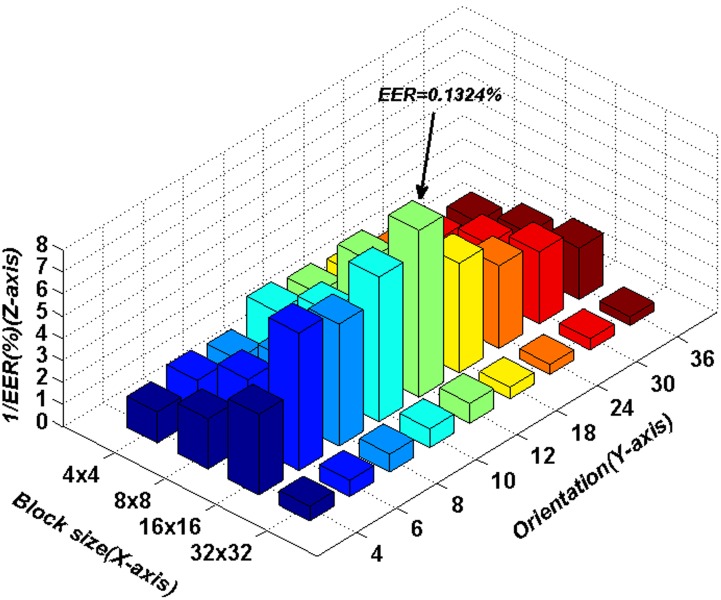
EER values using different parameters in the proposed method.

In the proposed VO–WRHOG method, block size (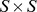) and orientation number (*N*) of the WRHOG are both important factors in improving recognition accuracy. Hence, blocks of different size (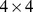, 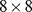, 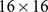, and 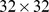) and different orientation number (*N* = 4, 6, 8, 10, 12, 18, 24, 30, and 36) were used in experiments in order to ascertain the optimal parameters to use for the blurred palmprint database. The experimental results are displayed using three-dimensional histograms in Fig. 9. In the figure, the *X*-axis and *Y*-axis represent the block size and orientation number, respectively. Note that we use 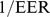 (and not EER) on the *Z*-axis for ease of viewing.

By considering the data in Fig. 9, some conclusions can be obtained, as follows. (1) For a fixed block size, as the orientation number is changed, the EER values around *N* = 12 correspond to minimal error. (2) For a fixed orientation number, the EER obtained using 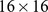 blocks is lower than those obtained using other block sizes. On the whole, regardless of the location on the *X*-axis and *Y*-axis, the distribution of the EER values appears similar to a convex function. It is also easy to obtain the extrema. Optimal recognition results are obtained using the appropriate block size and orientation number. Here, the block size required is 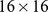 and the orientation number is 12. The EER with these choices had its lowest value (0.1324%).

### 4.2 Stability of the structure layer

**Figure 10:**
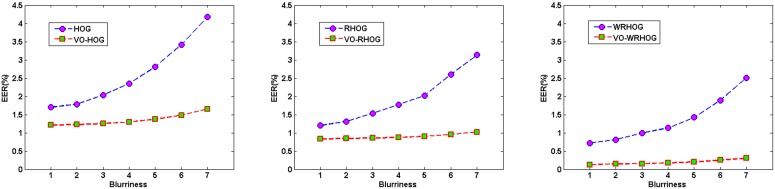
EER values obtained from palmprint images with different blurriness using different methods: (a) HOG and VO–HOG, (b) RHOG and VO–RHOG, and (c) WRHOG and VO–WRHOG.

To validate our conclusion in Section 2.3 that the structure layer is stable for palmprint images suffering different degrees of blurring, we designed a set of experiments to test the assertion. GDDM functions with different blurriness (or 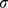 values, as mentioned in Section 2.3) were used to blur all the palmprint images in the PolyU database. Blurriness values of 1, 2, 3, 4, 5, 6, and 7, were used. Thus, palmprint databases with different amounts of blur were obtained. These were used to test the stability of our method. Fig. 10 shows the EER values for the palmprint images with different blurriness obtained using the HOG, VO–HOG, RHOG, VO–RHOG, WRHOG, and VO–WRHOG methods.

From the results in Fig. 10, it is obvious that for the PolyU palmprint databases with different amounts of blur, the recognition performance of the methods based on a structure layer are more stable than those methods which do not use a structure layer, and their EER values are subsequently lower. Therefore, the above experimental results and analysis demonstrate that the structure layer obtained via the VO decomposition model is stable for different degrees of blurring. At the same time, the proposed method based on the VO model is effective for blurred palmprint recognition.

### 4.3 Benefits of the proposed method

**Figure 11:**
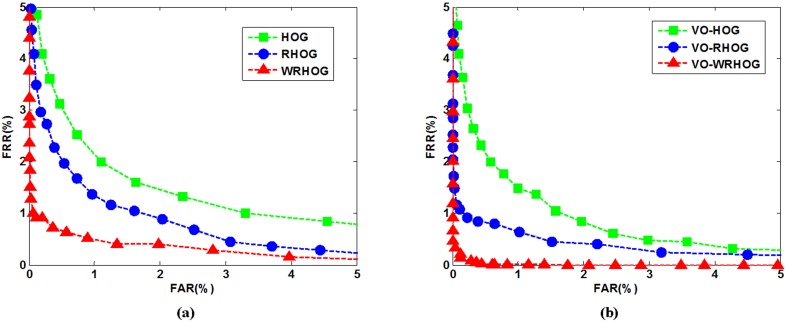
ROC curves obtained using different methods: (a) HOG, RHOG, and WRHOG, and (b) VO–HOG, VO–RHOG, and VO–WRHOG.

In our method, we propose using the RHOG method to improve robustness and we also consider fractal dimension as a weighting factor to improve the distinguishability between the characteristics. Hence, experiments were performed to illustrate the advantages of RHOG and fractal dimension in palmprint recognition. We separately used the HOG, RHOG, WRHOG, VO–HOG, VO–RHOG, and VO–WRHOG methods for the blurred PolyU palmprint database. Fig. 11 shows the ROC curves so obtained.

To manifest the benefits of the proposed method more clearly and more convincingly, we use a decidability index [34] as a measurement standard to evaluate the discrimination between genuine and imposter matching scores. This is defined as
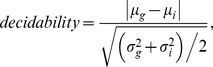
(29)where 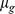 (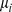) is the mean of the genuine (imposter) matching scores, and 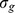 (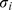) is the standard deviation of the genuine (imposter) matching scores. A large decidability index indicates strong distinguishability characteristics, i.e. high recognition accuracy or robustness, and *vice versa*. Table 1 lists the results obtained using the HOG, RHOG, WRHOG, VO–HOG, VO–RHOG, and VO–WRHOG methods in terms of EER and decidability index.

**Table 1:** Equal error rates and decidability indices corresponding to Fig. 11.

Method	EER (%)	Decidability index
HOG	1.7068	3.9868
RHOG	1.1979	4.4313
WRHOG	0.6222	5.6038
VO–HOG	1.3146	4.3366
VO–RHOG	0.7532	5.4099
VO–WRHOG	0.1324	6.6588

As can be seen from Fig. 11(a) and Table 1, the performance of the algorithms which do not use a VO model can be sorted in descending order as follows: WRHOG, RHOG, and HOG. Clearly, the decidability index of the RHOG method is larger than that of the HOG method, which indicates that the RHOG method has high robustness. Robustness embodies anti-interference performance with respect to translation, rotation, and noise. The gradient in the HOG method is sensitive to information variation in the image, and the resistance to rotation is also poor. As a consequence, HOG is not an appropriate method of blurred palmprint recognition. The RHOG method uses spatial region information to compute the gradient feature instead of the original gradient in the HOG method. This makes it more robust. By observing and analyzing the RHOG method, we find that although the RHOG method is robust and has got a qualified performance with respect to the orientation of the palmprint, it lacks good distinguishability because of image blur. Faced with this problem, we use fractal dimension as a weighting factor to improve the distinguishability of the characteristics. As listed in Table 1, the decidability index of the WRHOG method is considerably larger than those of the RHOG and HOG methods. In addition, we can reach the same conclusions from Fig. 11. From the experiment results and theoretical analysis, our method proves to be an effective and beneficial one.

### 4.4 Performance comparison with traditional algorithms

**Figure 12:**
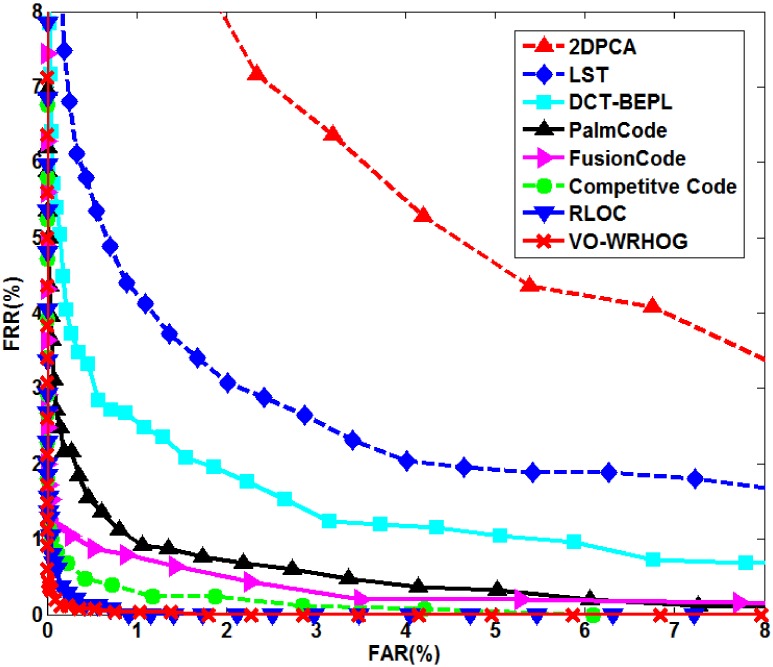
ROC curves for the high-performance and VO–WRHOG methods using data from the PolyU palmprint database.

**Figure 13:**
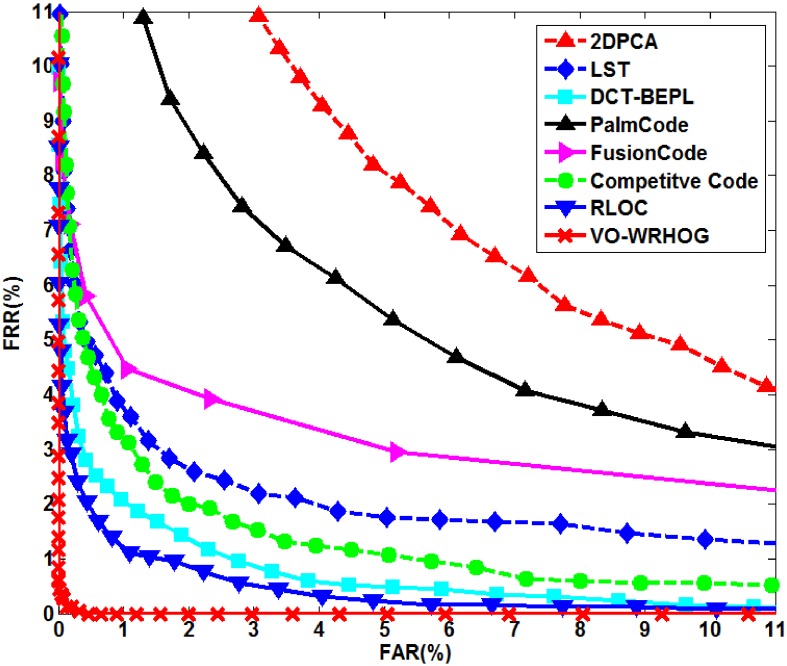
ROC curves for the high-performance and VO–WRHOG methods using data from the blurred PolyU palmprint database.

We performed experiments using the PolyU palmprint database and blurred PolyU palmprint database, and compared the results with some high-performance methods (2DPCA [13], LST [14], DCT–BEPL [11], PalmCode [15], FusionCode [16], Competitive Code [17], and RLOC [10]). Figs. 12 and 13 show the ROC curves for the high-performance and VO–WRHOG methods obtained using the PolyU and blurred PolyU palmprint databases, respectively. Table 2 also lists the EER values corresponding to Figs. 12 and 13.

**Table 2:** Comparison of the equal error rates obtained using other high-performance methods and the VO–WRHOG method.

Method	EER (%)	
	PolyU palmprints	Blurred-PolyU palmprints
2DPCA	5.2653	6.5943
LST	2.7208	2.4676
DCT–BEPL	1.9249	1.6173
PalmCode	0.9810	5.2653
FusionCode	0.8156	3.5215
Competitive Code	0.4684	2.0037
RLOC	0.1685	1.1149
VO–WRHOG	0.1421	0.1324

The texture of a palmprint can be effectively described using a texture-based coding method (e.g. PalmCode, FusionCode, Competitive Code, RLOC, etc.). We can see from Fig. 12 that these methods can achieve superior recognition accuracy using the PolyU palmprint database. However, when the palmprint image is blurred, the recognition accuracy is greatly diminished because of the loss of texture information (Fig. 13 and Table 2). The idea behind the 2DPCA, LST, and DCT–BEPL methods is to extract the stable features from the palmprint, which makes it possible to obtain a high robustness when recognizing blurred palmprints. However, the distinguishability of the features is not high using these methods, and the recognition result is not satisfactory. In contrast, the proposed VO–WRHOG method obtains good recognition results with both the PolyU and blurred PolyU palmprint databases.

Therefore, we can draw some conclusions from the analysis above: (i) Comparing EER values from the PolyU and blurred PolyU palmprint databases, the 2DPCA, LST, DCT–BEPL, and VO–WRHOG methods are more stable than the others. This means that these methods are appropriate for blurred palmprint recognition. (ii) The EER of the VO–WRHOG method was as low as 0.1324%, which is lower than the other methods. In other words, the VO–WRHOG method can achieve highly desirable recognition results with blurred palmprints.

### 4.5 Speed

The proposed method was implemented using MATLAB 2010a on a desktop PC with a modest CPU (2.90 GHz), and 2 GB of random access memory. The time consumed in each step of the VO–WRHOG method was recorded and is listed in Table 3. We considered 386 palmprint templates of the PolyU palmprint database or the blurred PolyU palmprint database as the gallery set. The total time consumed *T* can be computed as follows
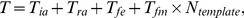
(30)where 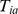, 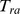, 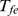, and 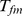 are the image acquisition time, ROI acquisition time, feature extraction time, and feature matching time, respectively. The quantity 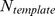 is the template number in the gallery set. Therefore, the total time consumed by our method, according to Eq. (30), is less than 1.3 s, which is short enough to meet the requirements for real-time recognition.

**Table 3:** The time consumed in each step.

Step	Time (ms)
Image acquisition [17]	<1000
ROI acquisition [17]	138
Feature extraction	119.8
Feature matching	0.059

## Discussion and Conclusions

In this paper, we proposed a VO–WRHOG method to solve the problem of blurred palmprint recognition. The experimental results demonstrate that blurred palmprints can be distinguished quite effectively by combining the use of the structure layer of the blurred image with the WRHOG method. We designed a series of experiments to gradually prove that the proposed method is of benefit for blurred palmprint recognition. First, the structure layer that is obtained using a VO model was proved to be a stable feature of the blurred palmprint image. Then, it was confirmed that the robustness of the RHOG method is superior to the HOG method’s. Finally, the distinguishability of the characteristics was improved by incorporating fractal dimension into the RHOG method.

In detail, our contributions are as follows:

1. Compared to previous methods, we used a VO decomposition model based on the optimal method of obtaining the structure layer which has stable and compact features. Hence, it is more effective than the DCT–BEPL [11], 2DPCA [13], and LST [14] methods.
2. The HOG method is used to extract orientation features. Unlike previous methods, HOG is an excellent spatial descriptor and can clearly depict the orientation information contained in a palmprint image. We improved the HOG method (RHOG) by making it more robust. The experimental results show that recognition accuracy is increased by using the RHOG method.
3. The novel idea that fractal dimension should be considered as a weighting factor is used (aimed at improving the distinguishability). Fractal dimension can measure the regularity of the information, and this is proved to be quite effective using a theoretical analysis. The experimental results are consistent with the theory.
4. Compared to previous high-performance palmprint recognition methods [10], [15]–[17], our method utilizes the stable information in the palmprint image to match characteristics, which makes the recognition results more stable when applied to the PolyU and blurred PolyU palmprint databases. At the same time, the method also produces excellent EERs and recognition speeds, which means that the proposed method outperforms all the previously-proposed high-performance methods.

In short, our proposed method successfully solves some of the troubling problems associated with blurred palmprint recognition very effectively.

## Supporting Information

Figure S1 ROC curves obtained using different methods: (a) HOG, RHOG, and WRHOG, and (b) VO–HOG, VO–RHOG, and VO–WRHOG.
(TIF)

Figure S2 ROC curves for the high-performance and VO–WRHOG methods using data from the PolyU palmprint database.
(TIF)

Figure S3 ROC curves for the high-performance and VO–WRHOG methods using data from the blurred PolyU palmprint database.
(TIF)

Table S1 Equal error rates and decidability indices corresponding to Fig. 11.
(DOC)

Table S2 Comparison of the equal error rates obtained using other high-performance methods and the VO–WRHOG method.
(DOC)
