# Supplementary figures and images for: Blurred Palmprint Recognition Based on Stable-Feature Extraction Using a Vese–Osher Decomposition Model
Source: PLoS One. 2014 Jul 3;9(7):e101866. doi: 10.1371/journal.pone.0101866 (PMC4081781; doi:10.1371/journal.pone.0101866)

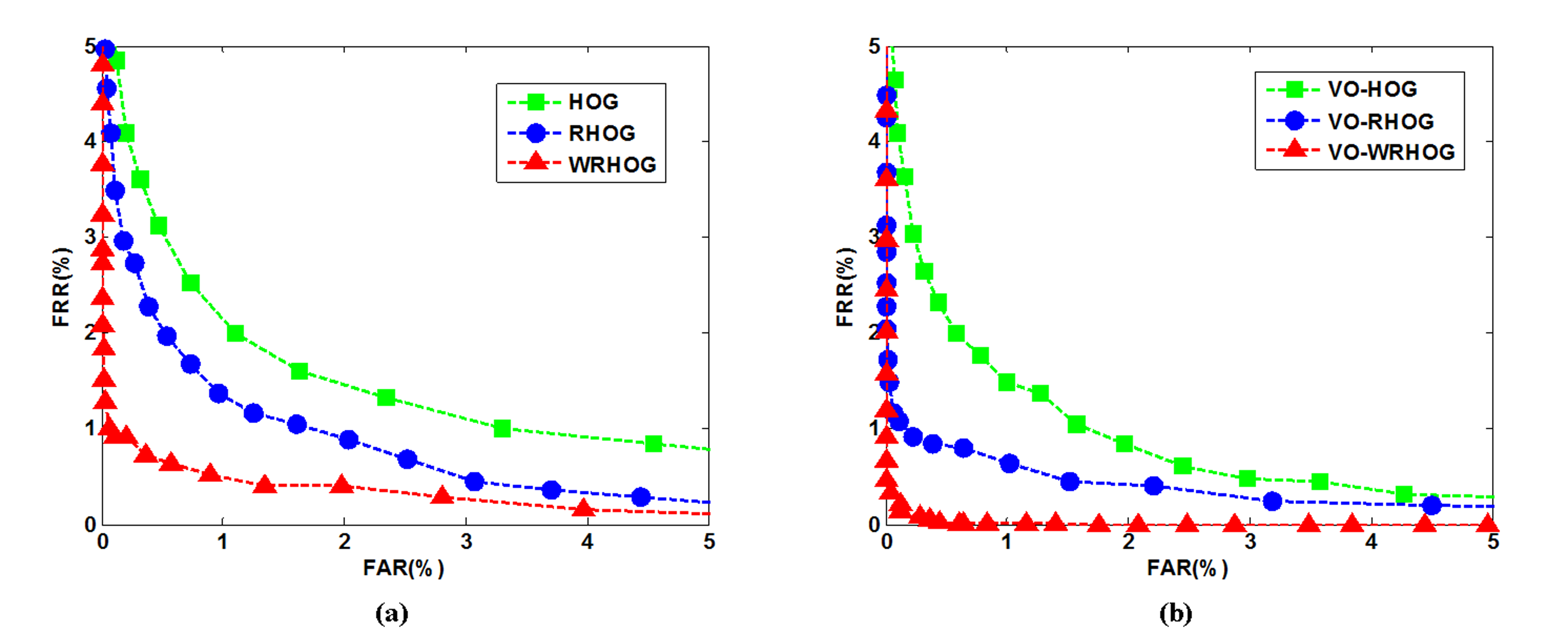

Supplement: Figure S1 — ROC curves obtained using different methods: (a) HOG, RHOG, and WRHOG, and (b) VO–HOG, VO–RHOG, and VO–WRHOG. (TIF) [file pone.0101866.s001.tif]

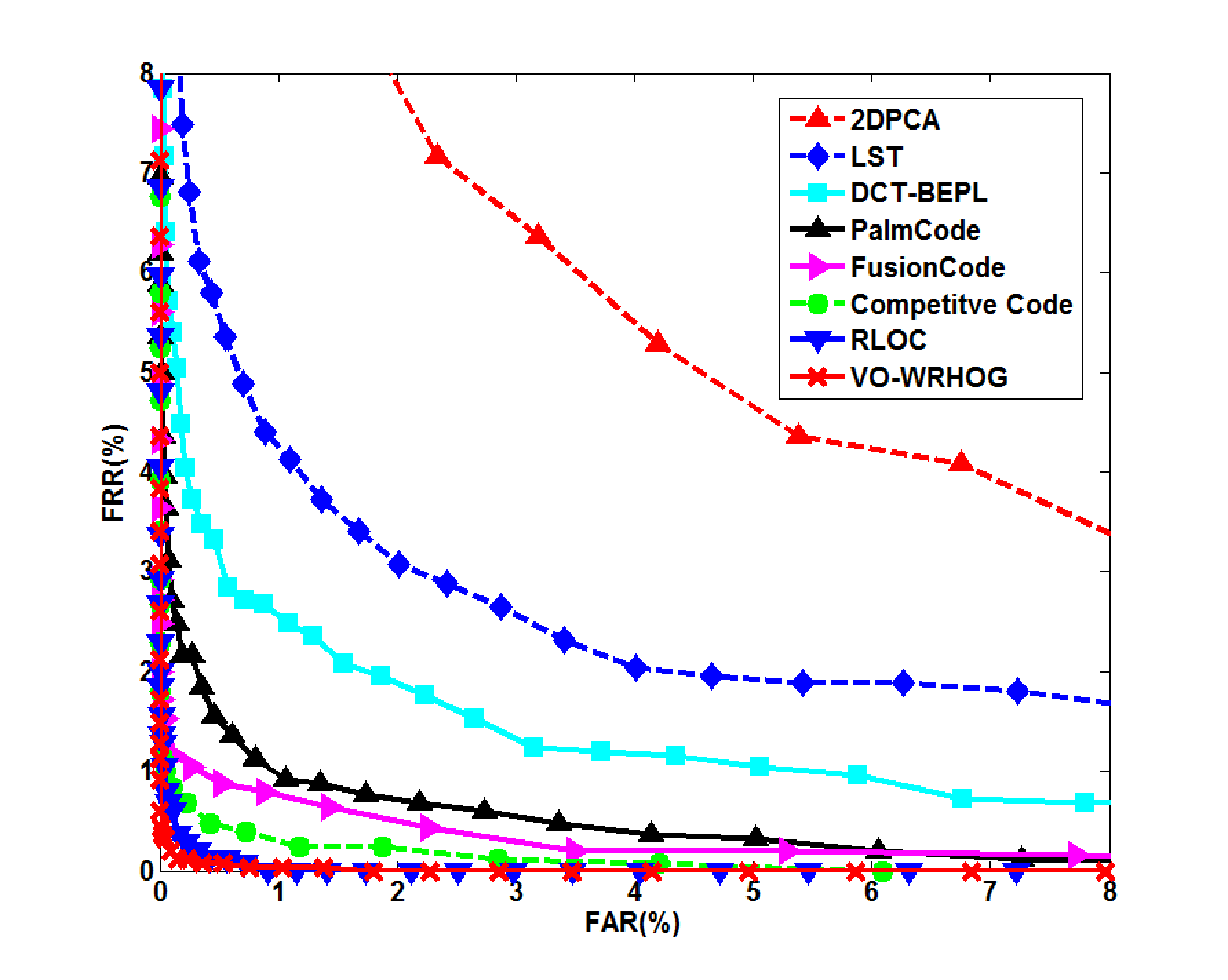

Supplement: Figure S2 — ROC curves for the high-performance and VO–WRHOG methods using data from the PolyU palmprint database. (TIF) [file pone.0101866.s002.tif]

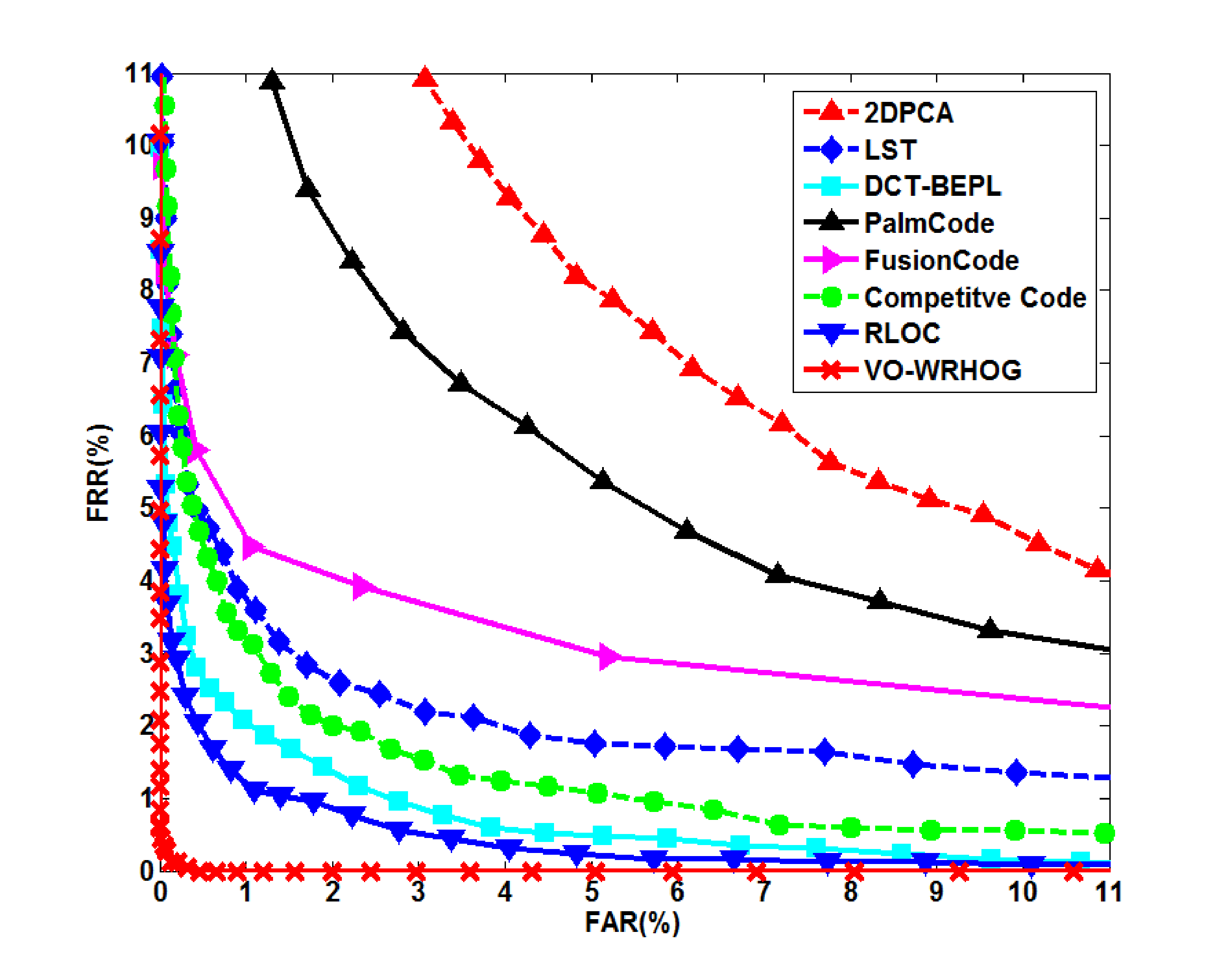

Supplement: Figure S3 — ROC curves for the high-performance and VO–WRHOG methods using data from the blurred PolyU palmprint database. (TIF) [file pone.0101866.s003.tif]
