# Supplementary material for: Blurred Palmprint Recognition Based on Stable-Feature Extraction Using a Vese–Osher Decomposition Model
Source: PLoS One. 2014 Jul 3;9(7):e101866. doi: 10.1371/journal.pone.0101866 (PMC4081781; doi:10.1371/journal.pone.0101866)
Supplement: Table S1 — Equal error rates and decidability indices corresponding to Fig. 11. (DOC) [file pone.0101866.s004.doc]

| **Table S1.** Equal error rates and decidability indices corresponding to Fig. 11. | | |
| --- | --- | --- |
| Method | EER (%) | Decidability index |
| HOG | 1.7068 | 3.9868 |
| RHOG | 1.1979 | 4.4313 |
| WRHOG | 0.6222 | 5.6038 |
| VO–HOG | 1.3146 | 4.3366 |
| VO–RHOG | 0.7532 | 5.4099 |
| VO–WRHOG | 0.1324 | 6.6588 |
