# Supplementary material for: Blurred Palmprint Recognition Based on Stable-Feature Extraction Using a Vese–Osher Decomposition Model
Source: PLoS One. 2014 Jul 3;9(7):e101866. doi: 10.1371/journal.pone.0101866 (PMC4081781; doi:10.1371/journal.pone.0101866)
Supplement: Table S2 — Comparison of the equal error rates obtained using other high-performance methods and the VO–WRHOG method. (DOC) [file pone.0101866.s005.doc]

| **Table S2.**Comparison of the equal error rates obtained using other high-performance methods and the VO–WRHOG method. | | |
| --- | --- | --- |
| Method | EER (%) | |
|  | PolyU palmprints | Blurred-PolyU palmprints |
| 2DPCA | 5.2653 | 6.5943 |
| LST | 2.7208 | 2.4676 |
| DCT–BEPL | 1.9249 | 1.6173 |
| PalmCode | 0.9810 | 5.2653 |
| FusionCode | 0.8156 | 3.5215 |
| Competitive Code | 0.4684 | 2.0037 |
| RLOC | 0.1685 | 1.1149 |
| VO–WRHOG | 0.1421 | 0.1324 |
